# Assessing the Recordkeeping Quality at the School of Dental Sciences, Universiti Sains Malaysia

**DOI:** 10.7759/cureus.55087

**Published:** 2024-02-27

**Authors:** Izzatul Najwa Abdul Rahman, Mohd Fadhli Khamis, Norhaslina Mohd Rokemi

**Affiliations:** 1 School of Dental Sciences, Health Campus, Universiti Sains Malaysia, Kubang Kerian, MYS

**Keywords:** practitioners, quality, crabel score, dental charting, dental records

## Abstract

Introduction: Dental records are an essential part of dental practice. The quality of dental recordkeeping is paramount in ensuring the delivery of high-quality dental care and is also important for medico-legal reasons. Should there be any dispute or need for review, detailed and well-maintained records can provide evidence of the care provided and the decision-making process.

Objective: The study aimed to assess the quality of dental recordkeeping and dental charting practice at the dental clinic School of Dental Sciences.

Methods: The study was conducted in a retrospective manner reviewing dental records of patients treated by specialists, dental officers, and postgraduate and undergraduate students at the Hospital Universiti Sains Malaysia over a five-year period. Eight key components of clinical dental records *i.e*. date of charting, legibility on the odontogram, no blank on the odontogram, whether any mistakes have been strikethrough and initials, medical history, dental history, investigation, and treatment plan were assessed. A modified CRABEL scoring system was used to assess the quality of data retrieved from dental records.

Results: The study involved the analysis of 324 case files. Among these, 90 files obtained scores ranging from 60% to 80%, with 7.7% attributed to undergraduates, 9.6% to dental officers, 6.8% to postgraduates, and 3.7% to specialists. The remaining 234 files achieved scores between 80% and 100%, with a breakdown of 17% from undergraduates, 15.4% from dental officers, 18.2% from postgraduates, and 21.3% from specialists.

Conclusion: Even though the overall quality of recordkeeping in this study is good, with most records achieving a CRABEL score of 80% and above, it's important to acknowledge that ideally, each component assessed should achieve a perfect score of 100%, as it will reflect the practitioners's work.

## Introduction

Maintaining an accurate dental record is critical for the sustained management of patient treatment. Proper documentation and charting are essential to adhere to the accepted standard of care [1]. A record is any item of information specifically related to a patient, regardless of its form or medium, and it is created or received by a practitioner, dental office, or any health-related institution, as part of providing care and conducting dental service [2]. Dental charting is a fundamental component of any dental record and constitutes an integral part of each dental examination. It provides a diagrammatic representation of a patient’s oral cavity that enables practitioners to assess various aspects, including the existing teeth, restorations, prostheses, bridges or implants, and any planned future treatment. Dental charting can be performed manually or digitally, with several software options available for dentists.

Keeping detailed dental records is important for dentists, not only to enhance patient care but also to protect themselves, as there are many rules and laws that highlight the necessity for proper documentation. In Malaysia, the Malaysian Dental Council (MDC) mandates that dental professionals maintain accurate and thorough patient records. The MDC has issued guidelines on dental recordkeeping and dental charting, serving as a reference for the expected standard of recordkeeping among practitioners. As of January 1, 2024, MDC enforces the mandatory practice of full mouth charting for dental professionals [2]. Therefore, it is crucial to produce and maintain precise dental records to ensure the delivery of high-quality patient care.

In many countries, there are reports of inaccuracies and incompleteness in dental records, as well as deviations from the expected standards [3-5]. These issues could have several implications, including impacts on patient care, legal problems, and challenges in identifying individuals in forensic cases. Maintaining accurate and complete dental records is essential for various reasons, including continuity of care, insurance claims, and legal documentation. The standardization of recordkeeping practices is also crucial to ensure that dental professionals adhere to the best practices and legal requirements.

While dental recordkeeping has been included in the undergraduate curriculum, the standard of quality in recordkeeping has not been verified. Therefore, the objective of this research is to assess the quality of dental recordkeeping and dental charting at the School of Dental Sciences using a modified CRABEL score of undergraduate students and benchmarking with other categories of practitioners.

## Materials and methods

A retrospective study of dental records was conducted from 2019 to 2023 at the Unit Rekod of Hospital Universiti Sains Malaysia (Hospital USM). Ethical approval was obtained from the Jawatankuasa Etika Penyelidikan Manusia (JePEM), USM/JEPeM/KK/23040313. The examination focused on the most recent entries in the case files, reviewing the dental charts for the date of charting, legibility on the odontogram, absence of blanks on the odontogram, identification of any strikethroughs of mistakes along with initials, and evaluation of medical history, dental history, investigation, and treatment plans. A method of stratified random sampling was used to select dental records from various practitioner groups, including specialists, dental officers, postgraduate students, and undergraduate students at the Hospital USM.

The data obtained was scored and rated using a modified version of the CRABEL scoring system developed by Crawford, Beresford, and Lafferty [6]. This scoring system is derived from the principles and guidelines established by the Royal College of Surgeons for medical records, originally intended for in-patients [7]. However, it has been modified and utilized in this study to evaluate scores based on the basic requirements for dental charting, as uncovered through a review of existing literature [8]. The CRABEL scoring system begins with a baseline score of 100%, and deductions are subtracted for any absent records. The modified CRABEL scoring system employed in this research is presented in Table 1.

**Table 1:** Modified CRABEL scoring system used to assess the adequacy of the dental records

Items	Marks
Date of charting	12.5
Legibility on the odontogram	12.5
No blank on the odontogram	12.5
Mistakes have been strikethrough and initials/no mistakes	12.5
Medical history	12.5
Dental history	12.5
Investigation	12.5
Treatment plan	12.5

The data were entered and analyzed using the Statistical Package for the Social Sciences (IBM SPSS Statistics for Windows, IBM Corp., Version 27.0, Armonk, NY). Descriptive statistics were presented as mean (SD) for continuous data and frequency in percentages for categorical data. Chi-square was employed to compare the degree of compliance among practitioners. The significance level was set at p<0.05.

## Results

During the study period, a total of 324 cases of patients' dental records were retrieved. The recording of patients' information was reviewed and assessed using the modified CRABEL score. One recording received a CRABEL score of less than 60%, and this was attributed to the undergraduate group. Ninety recordings received scores between 60% and 80%, with the highest frequency observed among dental officers at 9.6%, and the lowest among specialists at 3.7%. The majority of 234 files scored between 80% and 100%, with the highest frequency recorded for the specialists’ group at 21.3%, and the lowest among dental officers at 15.4%. The data is presented in Table 2.

**Table 2:** Frequency and CRABEL scores of practitioners Comparison between four groups using Chi-square p<0.05 is significant.

CRABEL scores (%)	Frequency (%)				
	Undergraduate students (n=81)	Dental officers (n=81)	Postgraduate students (n=81)	Specialists (n=81)	p-value
<20	-	-	-	-	-
20-40	-	-	-	-	-
40-60	1 (0.3%)	-	-	-	-
60-80	25 (7.7%)	31 (9.6%)	22 (6.8%)	12 (3.7%)	0.038
80-100	55 (17.0%)	50 (15.4%)	59 (18.2%)	69 (21.3%)	0.342

Among the eight items assessed individually, legibility on the odontogram and treatment plan received the highest score, with a frequency of 99.4%, while mistakes have been strikethrough, and initials or no mistakes recorded the lowest frequency at 67.9%. Table 3 provides a summary of these findings.

**Table 3:** Frequency of dental records with good full mouth charting practice

Items	Frequency (%)
Date of charting	87.0
Legibility on the odontogram	99.4
No blank on the odontogram	70.7
Mistakes have been strikethrough and initials/no mistakes	67.9
Complete medical history	98.5
Complete dental history	90.1
Investigation	89.2
Treatment plan	99.4
Total dental records 324	

## Discussion

This article aims to report on the quality of recordkeeping and dental charting at the Hospital USM by using the CRABEL score. Additionally, this study compared the results of the CRABEL scores among four groups of dental practitioners. The findings revealed that 69 out of 81 records among dental specialists achieved CRABEL scores ranging from 80% to 100%, indicating a high level of completeness and accuracy in their clinical documentation. This suggests that specialists, who generally have more years of experience and advanced training, maintain more thorough records compared to their less experienced counterparts.

Maintaining patient records is a primary responsibility of dentists, which is essential for safeguarding the interests of both the patient and the dental professional. In the United Kingdom, it is obligatory to establish and uphold complete and accurate records for their patients. Based on Principle 4 in the dental team standards document, dental professionals are obligated to promptly generate and keep detailed and precise records for each patient seen [9]. The General Dental Council mandates that all records documenting the work of dental practitioners must be understandable, precise, readable, and easily interpretable by anyone who may need to review them.

The MDC guidelines for dental recordkeeping and charting outline 10 key components [2]. These include personal information, chief complaint, comprehensive history (medical, dental, and social), clinical examination, investigations, diagnosis, treatment planning, consent, progress notes, and exit notes. Our study evaluated only a subset of these components. The assessed components received higher scores based on the CRABEL score. However, components such as the chief complaint, social history, consent, progress notes, and exit notes were not evaluated. Dental charting is an integral part of the clinical examination process. The guidelines outline several principles for dental charting. Charting should be conducted for all new patients at their initial visit, except for emergency cases, where it can be deferred to a subsequent visit. Re-charting is recommended after 12 months. An odontogram is required for charting, and every tooth must be included. Furthermore, radiographic findings should be documented in the dental charts.

Dental record inaccuracies and incompleteness in general, as well as failure to adhere to expected standards, have been reported on numerous occasions in several nations [3,4]. In the United Kingdom, Morgan found that only 70% of records had full tooth charts [4]. In Malaysia, few audits have been performed to evaluate dental records and charting. A study evaluated the impact of clinical audit training on dental students, and the findings indicated an improvement in the standards of dental records among clinicians during the second cycle audit [10]. In another study conducted by Azmi, it was found that among the domains investigated, the medical history and clinical examination had a lower mean percentage of completeness with 53.08% and 62% [11]. Compared to our study, a higher level of completeness for the medical history was achieved, at 98.5%. To maintain the quality of dental records, continuous audits play a vital role in cultivating constructive habits among clinicians and ensuring constant adherence to good clinical record standards.

The importance of complete and resourceful dental records in forensic odontology is undeniable, especially the dental charting component [12]. A comparison of postmortem and antemortem dental records, especially the charting, is essential for dental identification [13]. The information in dental charting like restorations and material used, appliances and prostheses, missing teeth, anomalies, and other pathologies is unique for human identification [14]. Blank odontogram or incomplete dental charting negatively affects the outcome of antemortem and postmortem comparisons.

The CRABEL scoring system was commonly used to assess the quality of medical recordkeeping [15,16]. It also had been used to evaluate dental recordkeeping [8,17]. The CRABEL score varies in different studies. In one of the studies conducted in Nigeria, the CRABEL score ranged from 65% to 95%. This scoring system has also been employed in their setting [17]. Nevertheless, the CRABEL score ranged from 10% to 100% in a study involving 100 undergraduate dental students in the United Kingdom [8]. It was determined that the CRABEL score was significantly below optimal. Among the recordings, only 42% of the records received a CRABEL score of 90% and above. The difference between the CRABEL scores reported by Pessian and Beckett in the above-mentioned study and those reported by Dosumu et al. may be attributable to the smaller sample size and assessment of fourth and fifth-year students as opposed to fifth and sixth-year students.

## Conclusions

In conclusion, the overall quality of recordkeeping and dental charting at Hospital USM, as assessed by the CRABEL score, is good. The results of this study can serve as a benchmark for future research. However, our study did not assess certain aspects of dental records such as the chief complaint, social history, consent, progress notes, and exit notes. Therefore, future studies should include all the key components outlined in the MDC guidelines.
